# Glucose-6-Phosphate Dehydrogenase Deficiency Presenting With Concurrent Hemolysis and Methemoglobinemia in a Young Woman: A Case Report of a Rare Medical Condition

**DOI:** 10.7759/cureus.94226

**Published:** 2025-10-09

**Authors:** Sri Amarnath Mathiyalagan, Archana Alva, Lunik Sarder

**Affiliations:** 1 Department of Emergency Medicine, Barking, Havering and Redbridge University Hospitals National Health Service (NHS) Trust, London, GBR

**Keywords:** ascorbic acid therapy, glucose-6-phosphate dehydrogenase (g6pd) deficiency, hemolytic anemia, hemolytic crisis, lyonization, methemoglobinemia, oxidative hemolysis, rare case report, sodium nitrite, x-linked enzymopathy

## Abstract

Glucose-6-phosphate dehydrogenase (G6PD) deficiency is the most common red-cell enzymopathy worldwide, yet symptomatic disease in women is uncommon because of its X-linked inheritance. The coexistence of G6PD deficiency with methemoglobinemia is exceedingly rare, with only isolated reports in the literature. This overlap poses a unique therapeutic dilemma because methylene blue, the standard treatment for methemoglobinemia, requires nicotinamide adenine dinucleotide phosphate generated via the G6PD pathway and is, therefore, contraindicated in people with G6PD deficiency. We describe the case of a young woman presenting with her first hemolytic crisis in whom previously undiagnosed G6PD deficiency was complicated by significant methemoglobinemia. Early recognition of this dual presentation allowed for prompt discontinuation of methylene blue, treatment of hemolysis and methemoglobinemia with transfusion and ascorbic acid, and subsequent recovery. This case illustrates the importance of recognizing G6PD deficiency in discordant oximetry-arterial blood gas scenarios and avoiding methylene blue, particularly when dietary nitrites are a potential precipitant.

## Introduction

Glucose-6-phosphate dehydrogenase (G6PD) deficiency is the most common enzymopathy worldwide, affecting an estimated 400 million people [1]. Its inheritance is X-linked, and while the condition predominantly affects hemizygous men, clinically significant disease in women is relatively uncommon because of lyonization and the presence of a second X chromosome [2]. Methemoglobinemia results from the oxidation of hemoglobin iron from the ferrous (Fe²⁺) to the ferric (Fe³⁺) state, impairing oxygen delivery to tissues despite adequate oxygen saturation [3]. Most cases are acquired, but congenital and secondary forms have been described. The coexistence of G6PD deficiency and methemoglobinemia is particularly rare [4-8]. Importantly, this overlap presents a distinct therapeutic challenge: methylene blue, the first-line agent for treating methemoglobinemia, requires nicotinamide adenine dinucleotide phosphate (NADPH) generated by the G6PD pathway, and its administration in G6PD-deficient patients can precipitate hemolysis [7]. We report a young female patient with an undiagnosed G6PD deficiency complicated by methemoglobinemia and hemolysis after likely nitrite exposure from processed meat.

## Case presentation

History and examination

A 32-year-old female patient of African descent presented to the emergency department with a two-day history of fatigue, yellowish skin discoloration, and passing dark colored urine. Her medical history includes gestational hypertension and Mounjaro (tirzepatide, a glucagon-like peptide-1 receptor analogue) usage for weight loss. There was no family history of medical conditions. She consumed beef jerky two days before the onset of symptoms. She was pale and icteric with otherwise normal systemic examination. Pulse oximetry showed peripheral oxygen saturation (SpO_2_) of 86%, but arterial blood gas (ABG) showed partial pressure of oxygen (pO_2_) of 11.4 kPa (≈85 mmHg). With supplemental oxygen of 15 L with a non-rebreather mask, SpO_2_ improved to 88%. She was neither tachypneic nor tachycardic, and her work of breathing was normal.

Investigation and treatment

Laboratory investigations included a complete blood count, liver and renal function tests, C-reactive protein, lactate dehydrogenase, gamma-glutamyl transaminase, methemoglobin level, and a direct antiglobulin (Coombs) test. Initial blood work demonstrated findings consistent with hemolysis along with elevated methemoglobin; the Coombs test was negative. Table 1 summarizes the hematological and biochemical parameters at presentation.

**Table 1:** The patient’s hematological and biochemical parameters at presentation G6PD: glucose‑6‑phosphate dehydrogenase; Hb: hemoglobin

Parameters	Results	Reference range
Hemoglobin	70 g/L	115-155 g/L
Reticulocyte %	4%	0.2-2.0%
Total bilirubin	197 µmol/L	1-21 µmol/L
Lactate dehydrogenase	476 IU/L	<250 IU/L
Methemoglobin	7.0%	<1.2%
G6PD activity	3.0 U/g Hb	5.0-12.1 U/g Hb
Peripheral smear	Blister cells and spherocytes	
Coombs test	Negative	

In view of the elevated methemoglobin level, she received methylene blue (1-2 mg/kg dosage). Shortly thereafter, her hemolysis worsened. The above-mentioned observation prompted hematology team evaluation, and confirmatory G6PD testing revealed severe enzyme deficiency. Methylene blue was discontinued, and the patient was subsequently managed with two units of packed red blood cells, ascorbic acid, and folic acid. She was discharged on day 6 with advice to avoid oxidative drugs and dietary triggers.

Over the course of treatment, hemoglobin initially declined to 61 g/L after methylene blue administration, accompanied by a reticulocyte count of 4.6%. Following transfusion, hemoglobin improved to 70 g/L with a reticulocyte rise to 6.0%, and it reached 115 g/L by four weeks, with normalization of methemoglobin levels. Overall, this represented a near 65% recovery in hemoglobin and complete resolution of methemoglobinemia, reflecting progressive marrow response and oxidative stabilization. Repeat G6PD testing at 12 weeks showed a level of 1.2 U/g Hb, confirming hereditary deficiency. Table 2 shows the evolution of her hemoglobin and reticulocyte count over time.

**Table 2:** Follow-up bloods

Timing	Hemoglobin	Reticulocyte %
After methylene blue on day 2	61 g/L	4.6%
After blood transfusion	70 g/L	6.0%
Four weeks later	115 g/L	0.5%

## Discussion

G6PD is an essential erythrocyte enzyme that is involved in the hexose monophosphate pathway to reduce reactive oxidative stress on the RBC membrane and its breakdown. Unless exposed to oxidative stress such as infection, drugs, or fava bean ingestion, most individuals are asymptomatic [7,8]. In patients with G6PD deficiency, exposure to certain oxidizing agents not only precipitates hemolysis but may also provoke the development of methemoglobinemia. Typical triggers include infectious illnesses, certain oxidant medications, particularly dapsone, sulfamethoxazole, and primaquine, and dietary exposure to fava beans [9]. The underlying mechanism is the reduction in NADPH availability, which impairs red cell antioxidant defense and promotes oxidative stress, thereby increasing methemoglobin levels [6,7].

In our patient, the absence of a family history, her young female sex, and the discordant finding of low peripheral oxygen saturation with a normal arterial PaO₂ initially suggested the possibility of primary methemoglobinemia, for which methylene blue would typically be the treatment of choice. However, the subsequent worsening of hemolysis following methylene blue administration redirected the diagnostic pathway toward underlying G6PD deficiency.

Identifying the precipitant is central to management. The patient denied infection or favism but reported consumption of beef jerky-processed meats commonly contain nitrates that are reduced to nitrites by intestinal flora [9,10]. Certain food items, such as refrigerated dim-sum, stuffed pork, and Chinese sausages, have also been implicated as potential sources of nitrates [10]. Nitrates and nitrites have been implicated in food-borne methemoglobinemia and can precipitate hemolysis in susceptible individuals [9-12]. Avoidance of oxidative triggers is, therefore, a key component of discharge counseling.

Although G6PD deficiency is X-linked, women may present with clinically significant hemolysis due to skewed X-chromosome inactivation (lyonisation). Bias toward the mutant allele renders the circulating red-cell pool predominantly deficient; under oxidative stress, inadequate NADPH production tips the balance toward hemolysis [9,13]. While large-scale reviews support genotyping as an adjunct to phenotypic testing in women with borderline enzyme activity, its primary role remains confirmatory rather than therapeutic [14]. Female presentations of oxidant-induced crises, including favism, are documented, underscoring that sex does not exclude the diagnosis [13].

Reports of concurrent G6PD-related hemolysis and methemoglobinemia in adults are uncommon but include cases precipitated by fava beans or other oxidants [4,6-8]. Our case adds to this literature by highlighting a likely dietary nitrite trigger, saturation gap as an early clue, and the importance of early hematology involvement in avoiding harmful therapies. More broadly, careful diagnostic reasoning is essential when hypoxemia on oximetry conflicts with normal ABG [9].

## Conclusions

This case brings forward the unusual and challenging coexistence of hemolysis and methemoglobinemia in a young female patient with previously undiagnosed G6PD deficiency. What made the journey even more complex was that methylene blue, usually the first-line therapy for methemoglobinemia, was given and led to worsening hemolysis. Paradoxically, this response became the very clue that pointed us toward the underlying diagnosis of G6PD deficiency. The case reminds us of the importance of looking beyond the obvious, especially when hypoxemia does not fit with ABG findings, and of always considering G6PD deficiency as a possibility, even in women. It also highlights that triggers may not always be classical like fava beans or infection but can be hidden in everyday exposures such as nitrate-containing foods. In the end, early recognition, prompt withdrawal of harmful therapy, and supportive interventions guided her recovery, underscoring how careful clinical observation and adaptable thinking remain central to managing rare and complex presentations.
